# Simultaneous Detection of Ochratoxin A and Aflatoxins in Industrial and Traditional Red and *isot* Pepper Flakes along with Dietary Exposure Risk Assessment

**DOI:** 10.1021/acsomega.2c02236

**Published:** 2022-08-26

**Authors:** Sebahat Oztekin, Funda Karbancioglu-Guler

**Affiliations:** † Department of Food Engineering, Faculty of Chemical and Metallurgical Engineering, Istanbul Technical University, Maslak, 34469 Istanbul, Turkey; ‡ Department of Food Engineering, Faculty of Engineering, Bayburt University, Bayburt 69000, Turkey

## Abstract

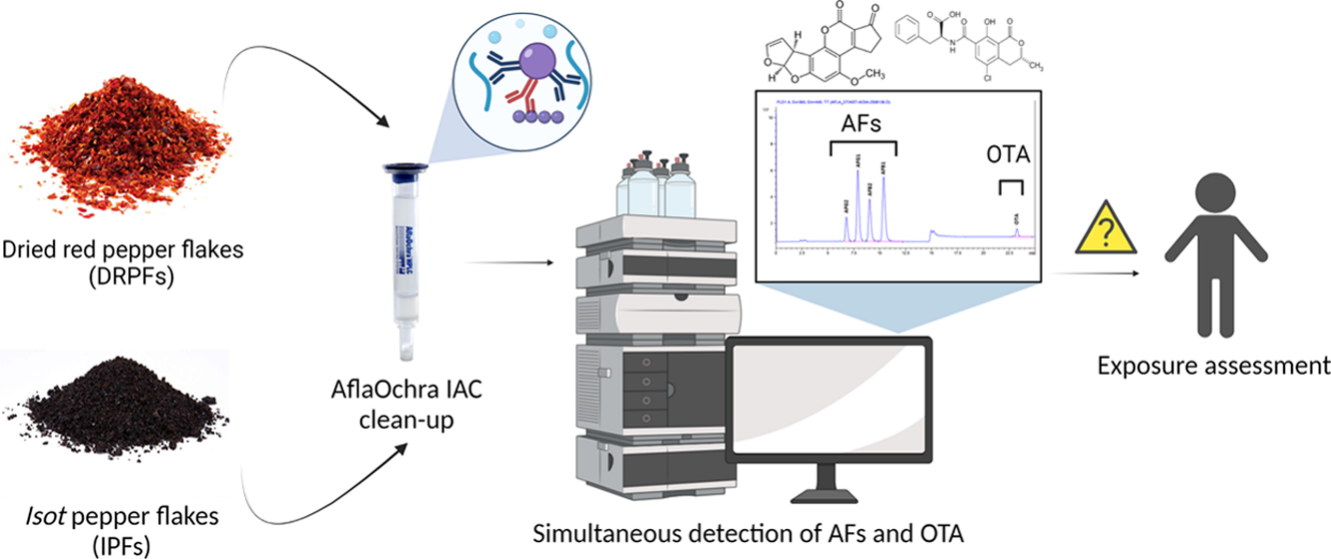

This study focused on the co-occurrence of aflatoxins (AFs) and ochratoxin A (OTA) in traditionally and industrially dried red pepper flakes (DRPFs) and *isot* pepper flakes (IPFs). Following the multitoxin immunoaffinity column (IAC) clean-up, high-performance liquid chromatography coupled with fluorescence detection (HPLC-FLD) was used to quantify AFs and OTA. The limit of detection (LOD) and limit of quantification (LOQ) values were 0.11 and 0.18 μg kg^–1^ (AFB_1_), 0.04 and 0.08 μg kg^–1^ (AFB_2_), 0.13 and 0.18 μg kg^–1^ (AFG_1_), 0.04 and 0.11 μg kg^–1^ (AFG_2_), and 0.10 and 0.21 μg kg^–1^ (OTA), respectively. AFB_1,_ AFB_2_, AFG_1_, and OTA were found to be positive in 93, 74, 17, and 94% of all samples, respectively. The contamination levels in positive samples ranged from 0.23 to 38.69, 0.04 to 2.14, 0.13 to 0.88, and 0.18 to 52.19 μg kg^–1^ for AFB_1_, AFB_2_, AFG_1_, and OTA, respectively, while no AFG_2_ was found above the detection limit (0.04 μg kg^–1^). None of the industrial *isot* peppers exceeded the European Union limits, while the levels of AFB_1_ (5 μg kg^–1^), total AFs (10 μg kg^–1^), and OTA (20 μg kg^–1^) of the traditional peppers were above the limit by 30% (16/54), 26% (14/54), and 4% (2/54), respectively. Co-occurrence of AFB_1_-AFB_2_-OTA was the most frequent, accounting for 54% (29/54) of all samples. At the upper bound (UB), estimated average exposure to AFB_1_, total AFs, and OTA was determined to be 0.175, 0.189, and 0.124 ng kg^–1^ bw day^–1^ in all samples, respectively. The margin of exposure (MoE) value of AFB_1_ and total AFs was found to be 977 and 909, indicating high health concerns compared to OTA (MoE >10,000). AFB_1_ and total AFs may result in 0.0058 and 0.0062 liver cancer cases/100,000 person/year at UB, respectively, and weekly OTA exposure was 0.868 ng kg^–1^ bw, well below the provisional tolerable weekly intake, hence not of health concern. AFs exposure could endanger health, whereas OTA posed no toxicological concerns through dried red pepper consumption

## 1 Introduction

Mycotoxigenic fungi (mostly *Aspergillus, Fusarium*, and *Penicillium*) can produce mycotoxins as secondary metabolites.^1^ Almost a quarter of crops were contaminated with mycotoxins annually, exposing nearly 4.5 billion people to mycotoxins through their daily diets.^2^ Mycotoxins have numerous health impacts that can be acute or mostly chronic, including cancer.^3^ Moreover, aflatoxins (AFs) were categorized as Group I by the International Agency for Research on Cancer (IARC) due to their highly toxic and carcinogenic effects on living organisms. Depending on the fluorescence color response under UV light, aflatoxins are divided into, B (blue; AFB_1_, AFB_2_) and G (green; AFG_1_, AFG_2_), with AFB_1_ being the most carcinogenic. Second, OTA was classified into Group 2B (possibly carcinogenic), a causative agent of the Balkan endemic nephropathy.^4^

Red peppers were categorized as paprika (nonpungent, sweet) and chili (pungent, hot) and represent any form of *Capsicum* spp.^5^ Red pepper (*Capsicum annuum* L. Solanaceae) was consumed freshly and mostly in dried form.^6,7^ Chili and pepper cultivation reached 1,990,926 ha worldwide, with a production value of 38,027,164 tons. Turkey ranked third after China and Mexico with 2,625,669 tons in 2019.^8^ Accordingly, the production of dried chili and pepper was 242,276 tons in Turkey.^9^

Dried red pepper flakes (DRPFs) could be produced in two ways: using traditional techniques as sun-dried or industrially as oven-dried.^10^ The majority of red peppers grown in the southeastern part of Turkey are classified as *isot* pepper flakes (IPFs), the traditional deep-red pepper. *Isot*, known as the traditional “Şanlıurfa pepper”, has also been geographically indicated.^9^*Isot* is darker in color, hotter in taste than ordinary dried red peppers, and turns black after sun-drying. To maintain its dark color, traditional *isot* production involves storing red peppers in plastic bags without direct exposure to sunlight.^6,10^

*Aspergillus* section *Flavi* and *Nigri* were found more frequently than *Penicillium* and *Fusarium* species in *Capsicum* spp.^7,11^ Mycotoxins can contaminate red peppers, particularly during harvesting, drying, processing, storage, and transportation.^7,12^ Many mycotoxins, including AFs (total AFs),^13,14^ OTA,^14^ zearalenone,^15^ fumonisins,^16^ deoxynivalenol,^17^ trichothecenes,^17^ citrinin,^15^ sterigmatocystin, and roquefortine C,^18^ were detected in red peppers. Furthermore, red pepper is a suitable substrate for a variety of mycotoxins; particularly, AFs and OTA are prevalent in red peppers.^13,14,17^ In this regard, the natural occurrence of AFs and OTA in red peppers was documented across the world, including Brazil,^14,19^ Chile,^13^ Greece,^20^ Indonesia,^21^ Lebanon,^16^ India,^22^ Italy,^17,23^ Nigeria,^24^ and South Africa.^18^ Therefore, research into mycotoxins in red peppers is critical for both domestic and international trade along with public health.

RASSF (Rapid Alert System for Food and Feed) has been ensuring food safety across Europe since 1979.^25^ During the 2020–2021 period, RASSF received 27 notifications on mycotoxins in chili, paprika, and capsicum peppers. Notably, 67% (18/27) of these were contaminated with AFs at unacceptable levels, representing 77% (14/18) of the chilies, placing them in the border rejection category. It is noteworthy that half of these chilies (9/18) originated from India. On the other hand, OTA (>20 μg kg^–1^) was found in 33% (5 chilies, 4 paprikas) of the peppers in the alert section, indicating a serious health risk. The presence of multiple mycotoxins in red peppers is still waiting to be addressed, and climate change may trigger the proliferation of mycotoxigenic fungi and subsequent multimycotoxin contamination.^26^ Given Turkey’s importance as a dried red pepper exporter,^8^ monitoring red pepper-related multiple mycotoxin exposure is critical. Furthermore, co-exposure to different combinations of mycotoxins could increase the cumulative risk more than exposure to a single type of mycotoxin alone.^27^

In this study, the lack of regulations on the co-occurrence of mycotoxins in *Capsicum* spp.^28,29^ led us to examine the co-occurrence rate of AFs-OTA in red peppers. Besides, IACs containing monoclonal antibodies were mainly employed in red peppers to detect mycotoxins individually.^7,30^ On the other hand, multimycotoxin IACs were used in several studies to extract AFs and OTA from red peppers.^14,31−33^ However, there are no reports on the use of multimycotoxin IACs to evaluate AFs and OTA in red peppers from Turkey. Instead, AFs and OTA extraction in red peppers was thoroughly investigated using monoclonal antibody-containing IACs.^30,34,35^ The use of multimycotoxin IAC can speed up the analysis and lower costs.^31^ Thus, the objectives of this research were: (i) to extract AFs and OTA from DRPFs and IPFs using multimycotoxin IAC with simultaneous detection, (ii) to compare current levels of AFs-OTA with acceptable limits, and (iii) to estimate dietary exposure and risk characterization through red pepper consumption in Turkey.

## 2 Results and Discussion

### 2.1 Method Performance

**Figure 1:**
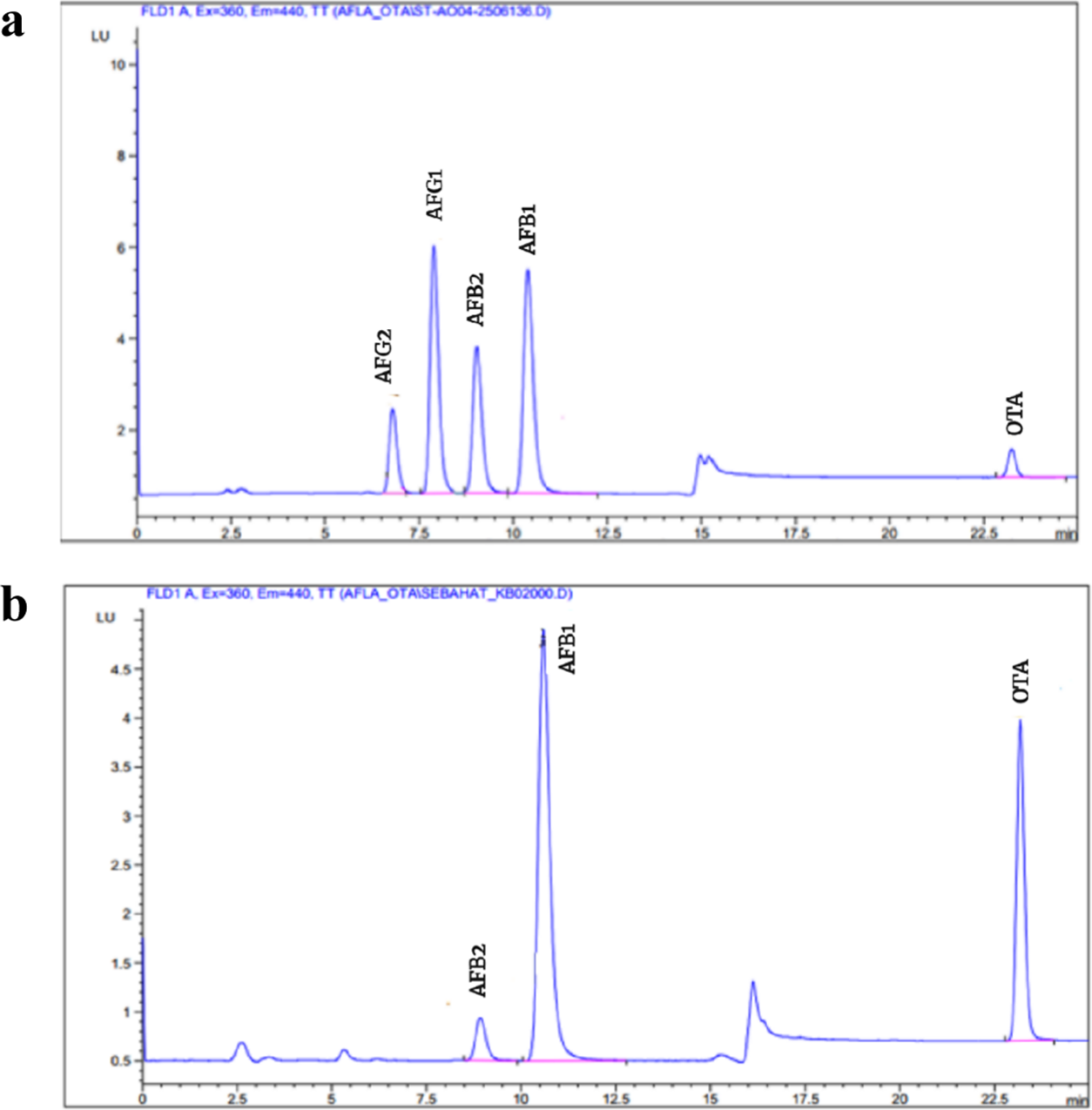
HPLC-FLD chromatograms. (a) AFs working standard solution 1.2 μg AFG_2_ L^–1^, 4 μg AFG_1_ L^–1^, 1.2 μg AFB_2_ L^–1^, 4 μg AFB_1_ L^–1^, and OTA (4 μg OTA L^–1)^. (b) Naturally contaminated traditional dried red pepper flakes (DRPFs) with 1.52 μg AFB_2_ kg^–1^, 29.92 μg AFB_1_ kg^–1,^ and 52.19 μg OTA kg^–1^.

AFG_2_, AFG_1_, AFB_2_, AFB_1_, and OTA were eluted in that sequence, with retention times of 6.96, 8.15, 9.11, 10.6, and 23.2 min, respectively. When the analytes were processed, no interference peaks appeared during the retention time, and the chromatograms showed good resolution. Figure 1a illustrates the chromatogram of standard solutions for AFs and OTA, while Figure 1b shows their natural occurrence in traditional DRPFs. All analytes had correlation coefficients greater than 0.98, indicating good linearity. The LODs and LOQs of the method are presented in Table 1, together with recoveries and repeatability values. Mean recoveries of AFs and OTA ranged from 63.8 to 89.8 and 75.8 to 87.0%, respectively. With good precision, RSD values for AFs and OTA ranged from 1.0 to 24.4 and 3.1 to 13.9%, respectively. Based on Commission Regulation (EC) No. 401/2006, the recoveries are adequate (between 50 and 120% for <1 μg kg^–1^ AFs, OTA with RSD <40% and between 70 and 110% for 1–10 μg kg^–1^ AFs, OTA with RSD <20%), except for AFG_2_ at 3 μg kg^–1^, which was less than 70%.^36^ Stroka et al.^37^ suggested that the recovery of AFG_2_ could not be less than 60% for a solution of 5 ng mL^–1^ toxin. Recently, when employing AflaOchra coloumn, Iha et al.^14^ found the lowest recovery rate for AFG_2_ in paprika, which was lower than the present findings (at the upper spike level of 5 ng g^–1^, recovery for AFG_2_ was determined to be 51%). Similarly, Hernandez Hierro et al.^33^ documented that at the 5 μg kg^–1^ spike level, recoveries for aflatoxins B (>97.8%) were higher than G types (>70.7%) and OTA (75.7%) in paprika samples extracted with the AflaOchra column. However, Brera et al.^23^ compared the performance of single- (AflaPrep and OchraPrep) and multimycotoxin (AflaOchraPrep) IACs in the recovery of paprika samples, which yielded the same (85, 84, and 70% for AFB_1_, AFB_2_, and AFG_2_) or even higher (from 75 to 78% for AFG_1_ and from 90 to 96% for OTA, respectively). In some cases, monoclonal IAC extraction of AFs resulted in lower recovery for AFG_2_ (<70%).^19^ Aligning with the current results, Trucksess et al.^38^ stated that IACs containing AFs-specific monoclonal antibodies had higher recovery rates than those containing both AFs-OTA-specific antibodies. In general, multimycotoxin IAC resulted in lower recoveries for AFG_2_.^14,31,33,38^ For example, Brera et al.^23^ extracted paprika samples with (0.1 and 10%) and without (only PBS) Tween 20 and concluded that PBS solution achieved the best results. Recently, Iha et al.^14^ recommended using PBS containing 0.5 and 1% of Tween 20 in the mycotoxin extraction from paprika and spices, respectively, resulting in the reduction of interfering peaks. Similarly, Palma et al.^13^ proposed adjusting the concentration of Tween 20–10% for improved AFs recovery. In the present work, 1% of Tween 20 in PBS was used as an extraction solvent, which needs to be optimized for the higher recovery of AFG_2_. Fortunately, the method’s performance was unaffected by the low recovery of AFG_2_.^4^

**Table 1:** Performance of the Analytical Method for *isot* and Dried Red Pepper Flakes^a^,^b^,^c^

			recovery values		
mycotoxins	LOD (μg kg^–1^)	LOQ (μg kg^–1^)	spiking level (μg kg^–1^)	recovery ± SD (%)	RSD (%)
AFB_1_	0.11	0.18	1	80.5 ± 17.3	21.4
			2	81.8 ± 1.4	1.7
			10	89.8 ± 4.3	4.8
AFB_2_	0.04	0.08	0.3	80.3 ± 17.8	22.2
			0.6	79.1 ± 3.0	3.8
			3	83 ± 2.8	3.4
AFG_1_	0.13	0.18	1	75.7 ± 18.5	24.4
			2	73.4 ± 2.8	3.9
			10	72.6 ± 1.1	1.5
AFG_2_	0.04	0.11	0.3	63.8 ± 1.1	1.7
			0.6	64.0 ± 0.9	1.4
			3	64.7 ± 0.6	1.0
OTA	0.10	0.21	0.5	75.8 ± 2.4	3.1
			1	87.0 ± 12.1	13.9
			3	85.3 ± 9.6	11.3

a SD = Standard deviation.

b RSD = Relative standard deviation = SD / mean × 100.

c LOD = Limit of detection, LOQ = Limit of quantification.

### 2.2 Presence of AFs and OTA in Red Pepper Samples

AFB_1_, AFB_2_, AFG_1_, and OTA were found to be positive in 93, 74, 17, and 94% of 54 samples tested, with mean contamination levels of 6.36, 0.30, 0.35, and 4.52 μg kg^–1^, and ranges of 0.23–38.69, 0.04–2.14, 0.13–0.88, and 0.18–52.19 μg kg^–1^, respectively, while AFG_2_ was not detected above the LOD of 0.04 μg kg^–1^. AFs and OTA were found in DRPFs and IPFs, mostly in higher frequencies but at lower concentrations (Figures S1a–c). Compared with their industrially processed counterparts, traditionally processed red peppers had higher mean levels for AFB_1_ (9.17 and 4.30 μg kg^–1^), AFs (9.77 and 4.62 μg kg^–1^), and OTA (6.95 and 1.69 μg kg^–1^), respectively (Table 2). Throughout industrial processing, peppers were dried at controlled temperature, air velocity, and humidity, resulting in minimal fungal growth and contamination.^10^ Moreover, adding salt increases sodium levels by suppressing fungal growth.^39^ In traditional processing, microenterprises dry the pepper as a whole pepper,^40^ resulting in a closed and high-humidity environment inside the pepper that favors fungal growth and increases mycotoxin levels..^41^ Another issue is food adulteration, which occurs when red peppers are sprinkled with water, resulting in a fungal infestation. Since elevated humidity facilitates water absorption and promotes the synthesis of mycotoxins, regular monitoring of humidity and refrigeration parameters was thought to be critical as a preventive measure.^42^ To prevent the occurrence of mycotoxins, unwounded chilies need to be carefully washed and dried following harvesting. When it comes to packaging, hermetic sealing helps to create an unfavorable environment for fungal proliferation.^40^

**Table 2:** Incidence and Concentration Levels of AFs and OTA in Traditional and Industrial *isot* Pepper Flakes (IPFs) or Dried Red Pepper Flakes (DRPFs)

			IPFs			DRPFs		all samples
mycotoxins	parameter	all IPFs (*n* = 13)	traditional (*n* = 11)	industrial (*n* = 2)	all DRPFs (*n* = 41)	traditional (*n* = 31)	industrial (*n* = 10)	*n* = 54
AFB_1_	positive samples,^a^*n* (%)	10 (76.9)	10 (90.9)	0 (0)	40 (97.5)	31 (100)	9 (90)	50 (93)
	range (μg kg^–1^)	0.23–38.69	0.23–38.69	n.d.^c^	0.20–37.95	0.28–37.95	0.2–2.52	0.23–38.69
	mean value (μg kg^–1^)	3.65	4.30	n.d.	7.22	9.17	1.17	6.36
	median value (μg kg^–1^)	0.56	0.64	n.d.	1.92	6.09	1.11	1.43
	no. samples >EU ML^d^ (%)	1 (7.7)	1 (9)	0 (0)	15 (36.5)	15 (48.3)	0 (0)	16 (29.6)
AFB_2_	positive samples, *n* (%)	8 (61.5)	8 (72.2)	0 (0)	32 (78)	28 (90.3)	4 (40)	40 (74)
	range (μg kg^–1^)	0.04–1.13	0.04–1.13	n.d.	0.04–2.14	0.04–2.14	0.04–0.07	0.04–2.14
	mean value (μg kg^–1^)	0.15	0.17	n.d.	0.35	0.45	0.03	0.30
	median value (μg kg^–1^)	0.04	0.06	n.d.	0.07	0.28	0.02	0.07
AFG_1_	positive samples, *n* (%)	1 (7.7)	1 (9.1)	0 (0)	8 (19.5)	7 (22.6)	1 (10)	9 (17)
	range (μg kg^–1^)	n.d.–0.77	n.d.–0.77	n.d.	n.d.–0.88	0.13–0.88	n.d.–0.17	0.13–0.88
	mean value (μg kg^–1^)	0.12	0.13	n.d.	0.11	0.12	0.08	0.35
	median value (μg kg^–1^)	n.d.	n.d.	n.d.	n.d.	n.d.	n.d.	0.07
AFG_2_	positive samples, *n* (%)	n.d.	n.d.	n.d.	n.d.	n.d.	n.d.	0 (0)
total AF^b^	positive samples, *n* (%)	10 (76.9)	10 (90.9)	0 (0)	40 (97.5)	31 (100)	9 (90)	50 (93)
	range (μg kg^–1^)	n.d.–40.61	0.33–40.61	n.d.	0.30–39.26	0.40–39.26	0.30–2.77	0.28–40.61
	mean value (μg kg^–1^)	3.93	4.62	n.d.	7.7	9.77	1.3	7.31
	median value (μg kg^–1^)	0.7	0.83	n.d.	2.06	6.49	1.22	6.8
	no. samples >EU ML (%)	1 (7.7)	1 (9)	0 (0)	13 (31.7)	13 (41.9)	0 (0)	14 (25.9)
OTA	positive samples, *n* (%)	11 (84.6)	9 (81.8)	2 (100)	40 (97.5)	31 (100)	9 (90)	51 (94)
	range (μg kg^–1^)	0.05–12.62	0.05–12.62	0.19–0.34	0.35–52.19	0.18–52.19	0.35–2.43	0.18–52.19
	mean value (μg kg^–1^)	1.47	1.69	0.27	5.48	6.95	0.91	4.52
	median value (μg kg^–1^)	0.5	0.66	0.27	2.57	3.21	0.69	1.95
	no. samples >EU ML (%)	0 (0)	0 (0)	0 (0)	2 (4.9)	2 (6.5)	0 (0)	2 (3.7)
	average *a*_w_	0.43	0.39	0.66	0.54	0.54	0.58	0.52

a Positive samples, *n* (%): mycotoxin level ≥ LOD.

b Total AF: sum of AFB_1_, AFB_2_, AFG_1_, and AFG_2._.

c n.d.: not detected, <LOD. The LODs for AFB_1_, AFB_2_, AFG_1,_ AFG_2_, and OTA are 0.11, 0.04, 0.13, 0.04, and 0.1 μg kg^–1^, respectively.

d EU ML: European Union maximum level (5 μg kg^–1^ for AFB_1_; 10 μg kg^–1^ for total AFs; 20 μg kg^–1^ for OTA).

The Turkish Food Codex adheres to European Commission Regulation No. 165/2010.^43^ In European Commission Regulation No. 165/2010,^28^ maximum permitted limits are only available for AFs and OTA in *Capsicum* spp. products (dried fruits thereof, whole or ground, including chilies, chili powder, cayenne, and paprika) with 5 μg kg^–1^ for AFB_1_ and 10 μg kg^–1^ for total AFs (sum of AFB_1_, AFB_2_, AFG_1_, and AFG_2_). The European Commission Regulation No. 2015/1137^29^ raised the maximum limit of OTA from 15 μg kg^–1^ to 20 μg kg^–1^. However, the regulations for foods containing multiple mycotoxins are not yet covered by European Union (EU) legislation. Furthermore, 30% (16/54), 26% (14/54), and 4% (2/54) of the samples surpassed the EU limit for AFB_1_, total AFs, and OTA, respectively. It should be highlighted that only traditional peppers surpassed the limits for total AFs (13/31 of DRPFs and 1/11 of IPFs), AFB_1_ (15/31 of DRPFs and 1/11 of IPFs), and OTA (2/31 of DRPFs). Furthermore, the present data revealed that aflatoxin B types were more frequently detected than their G counterparts. This could be explained by *Aspergillus flavus* and *Aspergillus parasiticus* contamination of dried chili.^21^ More recently, Ezekiel et al.^24^ reported that chili peppers were mostly associated with aflatoxigenic *A. flavus*. Likewise, Ozbey and Kabak^30^ reported that *A. flavus* was the most common fungi in red peppers, producing more aflatoxin B than *A. parasiticus*. Another study from Korea showed that 90% (9/10) of the *A. flavus* strains could produce AFs (146.88–909.53 μg kg^–1^) in ground red peppers.^44^

Over the last three decades, various studies have been undertaken to determine the levels of AFs and OTA in dried red peppers. Tosun and Ozden^35^ and Set and Erkmen^45^ focused on the levels of OTA and AFs in unpacked and packed dried red peppers from Turkey. The results pointed out that all unpacked peppers were contaminated with OTA between 1.1 and 31.7 μg kg^–1^, while packed counterparts had lower contamination within the range of 0.6–1.0 μg kg^–1^.^35^ Similarly, only unpacked peppers surpassed the limits for AFs and AFB_1_ by 17.1 and 23.1%, respectively, with values ranging from 0.087 to 97.4 and 0.067 to 89.99 μg kg^–1^, respectively.^45^ In this context, Bircan^46^ noted that all bazaar-sourced peppers surpassed the legal limits for AFs compared to supermarket-sourced equivalents in Turkey. The study indicated that paprika samples (27/30) had AFB_1_ (0.5–116.4 μg kg^–1^) and AFs (0.5–124.6 μg kg^–1^), while all chili powders (15/15) contaminated with AFB_1_ (1.6–80.4 μg kg^–1^) and AFs (1.8–85.9 μg kg^–1^), respectively. The results were found to be consistent with the existing data since unpacked dried red peppers were mostly sold in an open form on the streets and bazaars and were commonly produced by traditional techniques under uncontrolled conditions on the ground with sunlight.^35^ A similar situation was documented in Malaysia; Jalili and Jinap^47^ noted that 65 and 81.3% of 80 chilies contained AFs (0.2–79.7 μg kg^–1^) and OTA (0.2–101.2 μg kg^–1^), respectively, emphasizing that peppers from the open market had higher contamination levels than those from the supermarket. Accordingly, 25% of chili peppers from local and farmer’s markets in Nigeria exceeded the limit of AFs.^24^ In Korea, Ahn et al.^12^ detected lower OTA levels in mechanically dried paprikas than in sun-dried counterparts (0.82 and 3.83 μg kg^–1^, respectively). It should be highlighted that only traditional DRPFs and IPFs red peppers surpassed the acceptable limits by 42% (13/31) and 9% (1/11) for total AFs, and 48% (15/31) and 9% (1/11) for AFB_1_, respectively, while 6.5% (2/31) in traditional DRPFs exceeded the limits for OTA and by 48% (15/31) and 9% (1/11) in traditional DRPFs and IPFs for AFB_1_, respectively. Furthermore, the present data revealed that aflatoxin B types were more frequently detected than their G counterparts. This could be explained by *A. flavus* and *A. parasiticus* contamination of dried chili.^21^ More recently, Ezekiel et al.^24^ reported that chili peppers were mostly associated with aflatoxigenic *A. flavus*. Likewise, Ozbey and Kabak^30^ reported that *A. flavus* was the most common fungi in red peppers, producing more aflatoxin B than *A. parasiticus*. Another study from Korea showed that 90% (9/10) of the *A. flavus* strains could produce AFs (146.88-909.53 μg kg^–1^) in ground red peppers.^44^

### 2.3 Simultaneous Occurrence of AFs and OTA in Red Pepper Samples

AFB_1_-AFB_2_-OTA combination was detected in 23 out of 41 (56%) DRPFs and 6 out of 13 (46.2%) traditional IPFs. In other words, 27 out of 42 (64%) co-occurrences were from all traditional peppers (DRPFs + IPFs), while 17% (2/12) of them came from industrial DRPFs, accounting for 54% (29/54) of all samples. Importantly, neither co-occurrence nor exceedance of EU levels was detected in industrial IPFs. In addition, 17, 17, and 4% of 54 samples showed co-contamination patterns with AFB_1_-AFB_2_-AFG_1_-OTA, AFB_1_-OTA, and AFB_1_-AFB_2_, respectively (Figure S2). AFs-OTA contamination was found to be lower in IPFs than in DRPFs, especially in industrial types, which could be explained by the water activity,^48^ the type of peppers,^32,49^ drying temperature, and climate.^50^ Climate conditions in Turkey’s southeast area, which are warm and humid, may enhance fungus development in soil and air, placing red peppers at danger of mycotoxin accumulation while sun-drying on the ground.^45^ Water activity (a_w_) values ranged from 0.266 to 0.686 (Table 2), and IPFs had comparatively lower water activity values than DRPFs, which may help to reduce elevated mycotoxin levels. Supporting this evidence, Marín et al.^48^ stated that a_w_ values below 0.85 limit the growth of aflatoxigenic *A. flavus* in chili by 50%. The co-occurrence of AFs and OTA in red-scaled peppers has been documented across the world. In line with the current findings, Özbey and Kabak^30^ detected that 62.5% (15/24) of Turkey-based chili flakes and 41% (9/22) of chili powder samples had AFs-OTA co-occurrence, representing 52% (24/46) of all chilies. Exceedances of the limits for AFB_1_, AFs, and OTA in chili flakes were 16.7, 12.5, and 16.7%, while in chili powder, it was 13.6%, 4.5%, and 13.6%, respectively. They also noted that the red chili flakes and chili powder peppers with the highest OTA contamination (53.4 and 98.24 μg kg^–1^, respectively) also had the highest concentrations of AFB_1_ (11.45 and 35.77 μg kg^–1^, respectively). The present findings called attention that excessive OTA levels (25.86 and 52.19 μg kg^–1^, respectively) had the highest levels of AFB_1_ (37.95 and 29.92 μg kg^–1^, respectively).

Santos et al.^7^ reported that all *Capsicum* samples (17 paprika, 11 chilies, and 4 smoked paprika) contained AFs (1.8–83.7 μg kg^–1^) and OTA (4.3–474.7 μg kg^–1^) in peppers, along with deoxynivalenol (2 chilies and 1 paprika), zearalenone (4 chilies and 4 paprikas), and trichothecenes (2 chilies). Previously, these researchers found that 75% (48/64) of paprika and 65% (23/35) of chilies contained at least one type of mycotoxin, and the presence of OTA was associated with AFs.^5^

Recently, Iqbal et al.^42^ reported that 75.4 and 71% of 252 chili sauces tested positive for AFs and OTA, respectively, exceeding the limits of AFB_1_, total AFs, and OTA by 44.8, 42.1, and 23%, respectively, demonstrating the importance of monitoring mycotoxins in chili-derived products. Similarly, Motloung et al.^18^ detected AFB_1_ in 4/7 of paprika, 2/14 of coarse chili, and 4/4 of ground chili samples with concentrations ranging from 3 to 19, 8 to 11, and 7 to 8 μg kg^–1^, respectively. Moreover, the frequency of OTA was less than our results for 1/7, 1/14, and 3/4 of the corresponding samples. Finally, the simultaneous occurrence of AFB_1_ and OTA were determined in 1/4 of the ground chili.

The simultaneous presence of AFs-OTA in dried red peppers might be due to contamination with distinct fungi or one type of fungus that produces different mycotoxins.^18^ Furthermore, when one type of mycotoxin is detected, it is quite common for other types to occur in the same substrate.^51^ Mycotoxin cocktails can have synergistic or additive effects on living organisms.^52^ According to Sedmíková et al.,^53^ OTA may boost its mutagenic properties when AFB_1_ and OTA coexist in the same substrate.

### 2.4 Dietary Exposure Assessment and Risk Characterization

The exposure estimates for AFB_1_, total AFs, and OTA (0.174, 0.187, and 0.124 ng kg^–1^ bw day^–1^, respectively, at mean MB) are presented in Table 3. The higher the MoE value, the lower the exposure, especially when it exceeds the cutoff value of 10,000.^54^ It must be highlighted that no levels of exposure to AFB_1_ and OTA were judged safe, but the MoE was used to prioritize the risk management process.^27^ The MoE estimations for AFB_1_ and total AFs were 977 and 909, respectively This situation demonstrates a potential health risk associated with the consumption of dried red pepper, as well as the need to take precautions. Unlike, the MoE value for OTA was far higher than the threshold level of 10,000 in red peppers, indicating that it was not of health concern. The estimated weekly OTA exposure was derived by multiplying the daily exposure (0.124 ng kg^–1^ bw day^–1^) by 7, yielding 0.868 ng kg^–1^ bw week^–1^, which was dramatically lower than the PTWI of 100 ng kg^–1^ bw^55^ and 120 ng kg^–1^ bw,^56^ representing 0.87 and 0.73% of the corresponding PTWI values (Table 3). As a result, long-term co-exposure to AFs and OTA through dried red peppers may result in serious health effects.

**Table 3:** Dietary Exposure of AFB_1_, Total AFs, and OTA and Risk Assessment

concentration (μg kg^–1^)		exposure (ng kg^–1^ bw day^–1^)^e^			liver cancer risk (case/100000 persons)^f^	
MB^b^	LB^a^–UB^c^	MB^b^	LB^a^–UB^c^	MoE^d^	MB^b^	LB^a^–UB^c^
AFB_1_						
6.363	6.359–6.367	0.174	0.174–0.175	977	0.0057	0.0057–0.0058
total AFs						
6.792	6.711–6.887	0.187	0.184–0.189	909	0.0061	0.0060–0.0062
OTA						
4.515	4.512–4.521	0.124	0.123–0.124	>10000		

a LB (lower bound): results below the LOD were replaced with 0, and unquantified values (between LOD and LOQ) were replaced by LOD.

b MB (middle bound): results below the LOD were replaced with the value of LOD/2, and unquantified values (between LOD and LOQ) were replaced by LOQ/2.

c UB (upper bound): results below the LOD were replaced with LOD, and unquantified values (between LOD and LOQ) were replaced by LOQ.

d MOE, Margin of exposure, the ratio of benchmark dose, and the estimated intake of AFB_1_ and total AFs (170 ng kg^–1^ b.w day^–1^) or OTA (21.0 μg kg^–1^ b.w day^–1^) to MB of exposure.

e The average body weight for the Turkish adult population was estimated to be 72.8 kg, and all values were expressed in μg kg^–1^.

f Liver cancer risk, liver cancer cases/100,000 population/year.

Although there have been numerous studies on red pepper mycotoxins, little information is known on their dietary exposure and MoE. For example, in Sri Lanka, the MoE level of AFB_1_ in red chili (45-78) was far lower than current findings (977), showing that chilies are a major public health concern.^57^ A study on traditional Turkish *sürk* cheese (which contains various spices, including chili peppers) indicated that the MoE and EDI levels of AFB_1_ were 2982 and 0.057 ng kg^–1^ bw day^–1^, respectively. Since cheese has little amount of chili peppers which posed lower risks compared to present findings, while the EDI for OTA was 0.205 ng kg^–1^ bw day^–1^ with a PTWI value of 1.44, posing a higher risk (0.124 ng kg^–1^ bw day^–1^).^58^ In Pakistan, Iqbal et al.^42^ calculated the dietary exposure by employing the lowest and the maximum mean levels of mycotoxins instead of the substitution method. At the lowest concentration, the dietary exposure to AFB_1_, total AFs, and OTA in chili sauce samples were found to be 0.127, 0.337, and 0.417 ng kg^–1^ bw day^–1^, respectively, being higher than our exposure data except for AFB_1_. In another study, AFB_1_ exposure from Turkey-based dried red peppers was estimated to be 1.5 ng kg^–1^ bw day for a 20 g day^–1^ red pepper consumption,^59^ which was 9-fold higher than our findings. On the other hand, Tosun and Ozden^35^ observed that OTA exposure through dried red peppers was 0.3 ng kg^–1^ bw day^–1^ for 0.6 g day^–1^ consumption. More recently, Koutsias et al.^20^ found that AFB_1_ exposure in red pepper flakes was 0.02 ng kg^–1^ bw day.

According to Külahi and Kabak,^60^ OTA contamination occurred throughout the pre- and postharvest stages, at 0.181 μg kg^–1^ (MB estimate) in 34% (17/50) of chilies. Further, OTA exposure was detected to be 0.011 ng kg^–1^ bw week^–1^ (MB estimate), serving 0.01% of EFSA PTWI of 120 ng kg^–1^ bw and contributing to 0.4% of total OTA exposure. Apart from red peppers, the MoE values for AFB_1_ and AFs exposure in Turkey-based dried figs were detected to be 34,000 and 18,889, respectively, with 0.0002–0.0003 liver cancer cases/100,000 persons/year, showing negligible health concern (Oktay Basegmez, 2019). Dietary exposure and MoE values for AFB_1_ (0.04–0.12 ng kg^–1^ bw day^–1^, 1417–4250) and OTA (0.03–0.07 ng kg^–1^ bw day^–1^, >10,000) were estimated in Iran-based dried fruits (including mulberry, date, fig, and apricot), revealing that AFs but not OTA could raise health issues.^62^

In the present study, quantitative liver cancer risk results showed that AFB_1_ and total AFs were linked to 0.0057 and 0.0060 liver cancer cases/100,000 persons/year at LB estimate, respectively, which was far lower than that reported by Yogendrarajah et al.^57^ (0.046 and 0.028 cases/100,000 persons/year for AFB_1_ in North and South of Sri Lankan chilies, respectively). Due to differences in mycotoxin concentrations, consumption rates, and different food commodities, estimates of liver cancer risk can vary by country and region; for example, Huong et al.^63^ reported that rice and its products had the highest AFB_1_ (22 ng kg^–1^ bw day^–1^, 1.5 cases/100,000 persons, MoE = 8) and OTA (7.9 ng kg^–1^ bw day^–1^, MoE = 2674) exposure at MB estimate. The dietary exposure levels of AFB_1_ and AFs in hazelnuts were calculated as 0.016 and 0.023 ng kg^–1^ bw day^–1^, and for dried figs with 0.003 and 0.005 ng kg^–1^ bw day^–1^ (UB estimate), respectively, since the dietary contribution of hazelnuts was higher than that of dried figs.^64^ Similarly, Bol et al.^65^ investigated pasta and bakery products and observed the MoE (24.6) and EDI (6.9 ng kg^–1^ bw day^–1^) values of AFB_1_, indicating a substantial health risk, while OTA exposure was 4.88 ng kg^–1^ bw day^–1^, serving 30.5% of PTWI with negligible risk. In particular, nearly half of infants and children were exposed to at least two mycotoxins at elevated concentrations because of their low body weight.^66^ Accordingly, the populations with lower body weight were much more susceptible to mycotoxins, e.g., the average body weight in Vietnam was 50 kg.^63^ Furthermore, dietary exposure and risk assessments may vary by country or even region, which are also proportional to mycotoxin levels and consumption rates..^57,59^ Likewise, the results may differ among studies due to methodological differences, such as how the LOD and LOQ are determined and how left-censored data are processed (<LOD or <LOQ).^61^

## 3 Conclusions

The simultaneous detection of AFs and OTA in DRPFs and IPFs using multitoxin IAC clean-up was evaluated along with the dietary exposure risk assessment. With the exception of AFG_2_, the method’s performance in detecting AFs and OTA in dried red peppers was satisfactory. All industrially processed red peppers met the regulatory limits for AFB_1_, total AFs, or OTA. In contrast, traditional peppers exceeded the EU limits by 30% (16/54), 26% (14/54), and 4% (2/54), respectively, indicating the need for improved storage and drying settings in conjunction with good agricultural and manufacturing practices. Since traditionally processed DRPFs contained a higher frequency of AFs than OTA, their long-term consumption may raise the risk for health; thus, red peppers should be routinely controlled for mycotoxin levels to minimize consumer exposure. Among the co-occurrence patterns, AFB_1_-AFB_2_-OTA was the most frequent (54%) in all samples. Exposure to AFs and AFB_1_ (977 and 909, respectively) through red pepper consumption appeared to be of greater concern than OTA (MoE >10,000) for the adult population in Turkey. Risk assessments revealed that exposure to AFB_1_ and total AFs was associated with 0.0058 and 0.0062 liver cancer cases/100,000 persons/year (UB estimate). Weekly OTA exposure was 0.868 ng kg^–1^ bw week^–1^, which was far below the PTWI, hence not of health concern, accounting for 0.87 and 0.73% of the JECFA and EFSA PTWI values of 100 and 120 ng kg^–1^ bw, respectively. A thorough toxicological investigation is needed to establish limits on the concurrent exposure to multiple mycotoxins in dried red peppers and other foods.

## 4 Materials and Methods

### 4.1 Samples

Fifty-four red pepper flakes were collected, including 41 dried red pepper flakes (DRPFs, 31 traditional and 10 industrial) and 13 red-black *isot* pepper flakes (IPFs, 11 traditional and 2 industrial). DRPFs and IPFs were produced both industrially (mechanically dried, under controlled conditions) and traditionally (sun-dried, based on traditional techniques). Unfortunately, since IPFs were mostly produced by traditional methods, the source of industrially processed IPFs was limited. Samples were obtained randomly from various retail markets, herbal shops, bazaars, and local producers in various cities (Adana, Gaziantep, Kahramanmaraş, Şanlıurfa, Istanbul, and Ankara) throughout Turkey. Sampling was done following EC 401/2006, which states that the total sample weight for spices should be at least 500 g.^36^ For unpackaged samples (sold by weight), random sampling was performed to gather samples from diverse areas of a batch, and all pepper flakes were ground to a flake size of roughly 1–3 mm. Water activity (a_w_) was measured by a hygrometer (Novasina, LabTouch-a_w,_ Lachen, Switzerland) at 25 °C ± 1. Further, samples were kept at −18 °C and analyzed at room temperature.

### 4.2 Chemicals and Materials

The aflatoxin standard solution was supplied from Supelco (Bellefonte, PA; Aflatoxin B and G mix, Cat. No. 46304-U). In 1 mL of methanol, each standard mix contains 0.3 μg of AFG_2_, AFB_2_, and 1 μg of AFG_1_, AFB_1_. Aflatoxin standards (0.2–10.8 ng mL^–1^ for AFB_1_ and AFG_1_, and 0.06–3.24 ng mL^–1^ for AFB_2_ and AFG_2_) were dissolved in methanol:water (20:30, v/v). The OTA standard was purchased from Sigma-Aldrich (Steinheim, Germany), and working standard sets (0.1–5 ng OTA mL^–1^) were prepared in acetonitrile/water/acetic acid (99:99:2, v/v/v). The AflaOchraTest immunoaffinity column (Vicam, Watertown, MA) was employed in clean-up and preconcentration steps. All analytical and high-performance liquid chromatography (HPLC)-grade chemicals and reagents were supplied from Merck (Darmstadt, Germany). Phosphate-buffered saline (PBS) and phosphate buffer (PB) were prepared according to Trucksess et al.^67^

### 4.3 Aflatoxin and Ochratoxin A analysis

#### 4.3.1 Sample Extraction and IAC Clean-Up

Based on Trucksess et al. (2008) and Stroka et al. (2000), red peppers were extracted and cleaned up with slight modifications. According to combined methods, relevant mycotoxins were extracted and followed by AflaOchraTest IAC clean-up and HPLC-FLD with postcolumn derivatization. Samples (5 g) were mixed with 25 mL of methanol–0.5% sodium hydrogen carbonate (NaHCO_3_) (7:3, v/v) and 1 g of sodium chloride (NaCl) and shaken (Janke and Kunkel, IKA-Labortechnik KS 250) at 400 rpm for 10 min. Following centrifugation for 10 min at 8228*g* (Eppendorf Centrifuge 5804R, Hamburg, Germany), 7 mL of the upper phase was transferred to another tube. PB (28 mL) containing 1% Tween 20 was added to the tube and vortexed. Following glass microfiber filtration, AflaOchraTest was employed for 25 mL of the filtrate with 1–2 drops/s. Then, 5 mL of 10 mM PBS and pure water were used to wash the column; finally, 3 mL of air was passed through the column for the remaining drops. After 1 mL of methanol was used to elute bounded AFs and OTA from the column, 1 mL of pure water was added before analysis.

#### 4.3.2 HPLC-FLD Analysis

Agilent Technologies 1100 series with a quaternary pump, solvent distribution system, fluorescence detector (FLD), degasser, and Rheodyne injector with a 100 L loop was used for HPLC analysis. The Chemstation 3D solution program was used to process samples and collect data. In chromatographic separations, a 250 mm×4.6mm ODS-Hypersil C18 column (5 μm particle size; Hichrome Ltd., Reading, U.K.) was utilized. For postcolumn derivatization, CoBrA-cell (Vicam, Watertown) was employed, maintaining the column at 30 °C with a flow rate of 1 mL min^–1^. Mobile phase A (water:acetonitrile:methanol, 6:2:3, v/v/v with 350 μL of 4 M nitric acid, HNO_3_, and 120 mg of potassium bromide, KBr) and mobile phase B (acetonitrile:water:acetic acid, 99:99:2, v/v/v) were used for AFs and OTA under gradient conditions, respectively, at a flow rate of 1 mL min^–1^. Elution was conducted as follows: 100% mobile phase A for 12 min and 100% mobile phase B for an isocratic duration of up to 25 min. The wavelength of excitation (λ_Ex_) and emission (λ_Em_) was set to 360 and 440 nm for AFs (B_1_, B_2_, G_1_, and G_2_) until 12 min; 333 and 477 nm for OTA, respectively.^33^ For injection, the volume of samples and standards was set to 100 μL.

### 4.4 Analytical Quality Parameters

Linearity, sensitivity, recovery, and precision were assessed as a part of the method validation plan to determine AFB_1_, AFB_2_, AFG_1_, AFG_2_, and OTA in dried red pepper samples. Linearity was determined using seven-point calibration ranging from 0.2 to 10.8 ng mL^–1^ for AFB_1_ and AFG_1_, 0.06 to 3.24 ng mL^–1^ for AFB_2_ and AFG_2_, and 0.1 to 5 ng mL^–1^ for OTA, in triplicate. Linearity was calculated using peak area and analyte concentration. Linear regression analysis was performed to calculate the method’s linearity, which was represented as a correlation coefficient (*R*^2^).

Method’s sensitivity was expressed as detection (LOD) and quantification (LOQ) limits were derived according to recovery data,^68^ based on signal-to-noise (S/N) ratios of 3 and 10, respectively, with the following equations
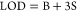

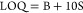
where “B” is the mean of blank samples and “S” is the sample standard deviation, with 10 injections.

Spiking was conducted using toxin-free blank red pepper samples containing certain concentrations of AFs (1, 2, and 10 μg kg^–1^ of AFB_1_ and AFG_1_; 0.3, 0.6, and 3 μg kg^–1^ of AFB_2_ and AFG_2_) and OTA (0.5, 1, and 3 μg kg^–1^) used for method’s accuracy. All samples were brought to room temperature overnight before extraction. Spiking was conducted in six replicates. Following HPLC-FLD quantification, the final mycotoxin content in a spiked sample (as explained in Sections 4.3.1 and 4.3.2) was compared to the known initial concentration to estimate recoveries. The precision was calculated at three different concentrations through a six-replicated analysis of spiked samples on the same day and expressed as the percent relative standard deviation (RSD) of the replicate measurements.

### 4.5 Dietary Exposure and Risk Assessment

#### 4.5.1 Margin of Exposure (MoE) Approach

For the calculation of dietary exposure, the margin of exposure (MoE) approach was used, which was a fraction of the BMDL_10_ (benchmark dose lower confidence limit 10) and estimated daily intake (EDI) of AFB_1_ or OTA, where BMDL_10_ is the lowest dose to cause a 10% increase in cancer incidence in rodents.^69^ Considering the risks of liver and renal cancer, the BMDL_10_ value for AFB_1_ and OTA was estimated to be 170 ng kg^–1^ bw day^–1^,^54^ and 21 μg kg^–1^ bw day^–1^,^56^ respectively. EDI level was calculated using average toxin concentrations via the substitution method.^70^ Thus, the values under LOD were substituted with 0, LOD/2, and LOQ to calculate lower (LB), middle (MB), and upper bound (UB) levels, respectively.^61−63^ Per capita consumption of dried red peppers was considered 2 g day^–1^, and the average body weight (bw) for Turkish adults was approximately 72.8 kg.^9^ The dietary exposure was determined by multiplying the contamination level per body weight with the dietary intake.^62^ EDI value was calculated as shown in eq 1
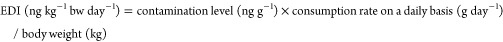
1

#### 4.5.2 Risk Assessment

Given that the MoE score indicates the degree of concern for mycotoxin exposure, an additional risk assessment is required to detect the risk of liver cancer.^69^ People who tested positive (PHBsAg^+^) and negative (PHBsAg^–^) for hepatitis B surface antigen (eq 2) were included in calculations, and consuming AFB_1_-contaminated foods was linked with a 30-fold increase in the risk of getting liver cancer.^3^ Hepatitis B virus infection was classified as intermediate endemicity (2–8%) or high (> 8%) by the World Health Organization,^71^ and HbsAg prevalence was reported to be 6.2 and 8.2% in the urban and rural areas, respectively, in Turkey’s southeastern region.^61,72^ Therefore, the risk of liver cancer was computed under the worst-case scenario, with an assumed population value of 8%. The risk of AFB_1_-related liver cancer was calculated by multiplying the EDI and the average carcinogenic potency (*P*_cancer_) (eq 3).^63^
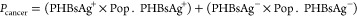
2
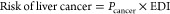
3PHBsAg^+^ = 0.3 cancers/year/100,000 population ng^–1^ AFB_1_ kg^–1^ bw day^–1^; PHBsAg^–^ = 0.01 cancers/year/100,000 population ng^–1^ AFB_1_ kg^–1^ bw day^–1^; Pop. PHBsAg^+^ = proportion of population positive with Hepatitis B (0.08); Pop. PHBsAg^–^ = proportion of population negative with Hepatitis B (0.92); *P*_cancer_ = 0.3 × 0.08 + 0.01 × 0.92 = 0.033.

AFB_1_ exposure (even at relatively low levels, ≤1 ng kg^–1^ bw day^–1^ in developed countries) can contribute to liver cancer.^73,74^ AFB_1_ has no safe level because of its severe carcinogenic and genotoxic effects; hence, it should be “as low as reasonably achievable (ALARA)” to protect public health.^75^ The average potency for liver cancer from eq 3 was calculated as 0.033, which was utilized in the present risk estimations (Table 3). However, Kuiper-Goodman^76^ calculated the provisional maximum tolerable daily intake (PMTDI) for AFB_1_ (1 ng kg^–1^ bw day^–1^). Therefore, for non-European countries, exposing 1 ng kg^–1^ bw day^–1^ of AFB_1_ would cause liver cancer by 0.083 cases/100,000 persons/year.^3^ As a result, the risk of liver cancer in Turkey was recalculated using the value 0.083 (Risk of liver cancer = 0.083 × EDI of AFB_1_), yielding 0.0144,0.0145, and 0.0146 cases/100,000 persons/year for MB, LB, and UB exposure levels, respectively, which were higher than the current estimates. However, for OTA, provisional tolerable weekly intake (PTWI) was set as 100 ng kg^–1^ bw^55^ and 120 ng kg^–1^ bw,^56^ respectively. Further, estimated OTA exposure was compared to the PTWI value to determine the risk assessment; exceeding the PTWI posed a risk.

## Supporting Information Available

Experimental details; contamination ranges of the AFs and OTA, and co-occurrence rates of their combinations in red pepper samples (PDF)

## Abbreviations Used

AFs: aflatoxins
AFB_1_: aflatoxin B_1_
AFB_2_: aflatoxin B_2_
AFG_1_: aflatoxin G_1_
AFG_2_: aflatoxin G_2_
DRPFs: dried red pepper flakes
EDI: estimated daily intake
EFSA: European Food Safety Authority
FAO: Food and Agricultural Organization of the United Nations
HPLC-FLD: high-performance liquid chromatography-fluorescence detector
IARC: International Agency for Research on Cancer
IAC: immunoaffinity column
IPFs: *isot* pepper flakes
LB: lower bound
LOD: limit of detection
LOQ: limit of quantification
MB: middle bound
MoE: margin of exposure
OTA: ochratoxin A
PTWI: provisional tolerable weekly intake
RASSF: rapid alert system for food and feed
RSD: relative standard deviation
TWI: tolerable weekly intake
UB: upper bound

## References

[ref1] Ostry V.; Malir F.; Toman J.; Grosse Y. Mycotoxins as Human Carcinogens—the IARC Monographs Classification. Mycotoxin Res. 2017, 33, 65–73. 10.1007/s12550-016-0265-7. 27888487

[ref2] Eskola M.; Kos G.; Elliott C. T.; Hajšlová J.; Mayar S.; Krska R. Worldwide Contamination of Food-Crops with Mycotoxins: Validity of the Widely Cited ‘FAO Estimate’ of 25%. Crit. Rev. Food Sci. Nutr. 2020, 60, 2773–2789. 10.1080/10408398.2019.1658570. 31478403

[ref3] FAO/WHO. World Health Organization & Food and Agriculture Organization of the United Nations. Evaluation of Certain Food Additives and Contaminants: Forty-Ninth Report of the Joint FAO/WHO Expert Committee on Food Additives. World Heal. Organ., 1999. https://apps.who.int/iris/handle/10665/42142. 10079756

[ref4] IARC. International Agency for Research on Cancer Monographs on the Evaluation of Carcinogenic Risks to Humans. Some Naturally Occurring Substances: Food Items and Constituents, Heterocyclic Aromatic Amines and Mycotoxins, Vol. 56; World Heal. Organ., 1993.

[ref5] Santos L.; Marín S.; Sanchis V.; Ramos A. J. Co-Occurrence of Aflatoxins, Ochratoxin A and Zearalenone in *Capsicum* Powder Samples Available on the Spanish Market. Food Chem. 2010, 122, 826–830. 10.1016/j.foodchem.2010.03.070.

[ref6] Hayoglu I.; Didin M.; Turkoglu H.; Fenercioglu H. The Effects of Processing Methods on Some Properties of Hot Red and Red-Blackish Ground Peppers. Pakistan J. Biol. Sci. 2005, 8, 1420–1423. 10.3923/pjbs.2005.1420.1423.

[ref7] Santos L.; Marín S.; Mateo E. M.; Gil-Serna J.; Valle-Algarra F. M.; Patiño B.; Ramos A. J. Mycobiota and Co-Occurrence of Mycotoxins in *Capsicum* Powder. Int. J. Food Microbiol. 2011, 151, 270–276. 10.1016/j.ijfoodmicro.2011.09.011. 21993449

[ref8] FAO. Food and Agricultural Organization of the United Nations. FAO Statistical Databases and Data Sets. Food Agric. Organ., 2020. http://www.faostat.fao.org/.

[ref9] TSI. Turkish Statistical Institute. The Summary of Agricultural Statistics. Turkish Stat. Inst., 2020. http://tuik.gov.tr.

[ref10] Korkmaz A.; Hayaloglu A. A.; Atasoy A. F. Evaluation of the Volatile Compounds of Fresh Ripened *Capsicum annuum* and Its Spice Pepper (Dried Red Pepper Flakes and Isot). LWT 2017, 84, 842–850. 10.1016/j.lwt.2017.06.058.

[ref11] Chuaysrinule C.; Maneeboon T.; Roopkham C.; Mahakarnchanakul W. Occurrence of Aflatoxin- and Ochratoxin A-Producing *Aspergillus* Species in Thai Dried Chilli. J. Agric. Food Res. 2020, 2, 100054 10.1016/j.jafr.2020.100054.

[ref12] Ahn J.; Kim D.; Jang H.; Kim Y.; Shim W.-B.; Chung D.-H. Occurrence of Ochratoxin A in Korean Red Paprika and Factors to Be Considered in Prevention Strategy. Mycotoxin Res. 2010, 26, 279–286. 10.1007/s12550-010-0067-2. 23605491

[ref13] Palma P.; Godoy M.; Vidal M.; Rivera A.; Calderón R. Adaptation, Optimization, and Validation of a Sensitive and Robust Method for the Quantification of Total Aflatoxins (B1, B2, G1, and G2) in the Spice Merkén by HPLC-FLD with Post-Column Derivatization. Microchem. J. 2022, 178, 107342 10.1016/j.microc.2022.107342.

[ref14] Iha M. H.; Rodrigues M. L.; Trucksess M. W. Multitoxin Immunoaffinity Analysis of Aflatoxins and Ochratoxin A in Spices. J. Food Saf. 2021, 41, e12921 10.1111/jfs.12921.

[ref15] Li M.; Tong Z.; Gao X.; Zhang L.; Li S. Simultaneous Detection of Zearalenone, Citrinin, and Ochratoxin A in Pepper by Capillary Zone Electrophoresis. Food Addit. Contam. Part A 2020, 37, 1388–1398. 10.1080/19440049.2020.1769197. 32546103

[ref16] El Darra N.; Gambacorta L.; Solfrizzo M.; Gambacorta L.; Solfrizzo M. Multimycotoxins Occurrence in Spices and Herbs Commercialized in Lebanon. Food Control 2019, 95, 63–70. 10.1016/j.foodcont.2018.07.033.

[ref17] Gambacorta L.; Magistà D.; Perrone G.; Murgolo S.; Logrieco A. F.; Solfrizzo M. Co-Occurrence of Toxigenic Moulds, Aflatoxins, Ochratoxin A, Fusarium and Alternaria Mycotoxins in Fresh Sweet Peppers (Capsicum annuum) and Their Processed Products. World Mycotoxin J. 2018, 11, 159–173. 10.3920/WMJ2017.2271.

[ref18] Motloung L.; De Saeger S.; De Boevre M.; Detavernier C.; Audenaert K.; Adebo O. A.; Njobeh P. B. Study on Mycotoxin Contamination in South African Food Spices. World Mycotoxin J. 2018, 11, 401–409. 10.3920/WMJ2017.2191.

[ref19] Iha M. H.; Rodrigues M. L.; de Cássia Briganti R. Survey of Aflatoxins and Ochratoxin A in Spices from Brazilian Market. Braz. Arch. Biol. Technol. 2021, 64, 0–8. 10.1590/1678-4324-2021210244.

[ref20] Koutsias I.; Kollia E.; Makri K.; Markaki P.; Proestos C. Occurrence and Risk Assessment of Aflatoxin B1 in Spices Marketed in Greece. Anal. Lett. 2021, 54, 1995–2008. 10.1080/00032719.2020.1832509.

[ref21] Wikandari R.; Mayningsih I. C.; Sari M. D. P.; Purwandari F. A.; Setyaningsih W.; Rahayu E. S.; Taherzadeh M. J. Assessment of Microbiological Quality and Mycotoxin in Dried Chili by Morphological Identification, Molecular Detection, and Chromatography Analysis. Int. J. Environ. Res. Public Health 2020, 17, 1847 10.3390/ijerph17061847. PMC7143392 32178381

[ref22] Hadil mon V.; John Kennedy Z.; Paranitharan V.; Karthikeyan S. Mycotic Contamination and Aflatoxin Potential of Molds in *Capsicum annum* (Chili), and Chili Powder Commercialized in South Indian Markets. Toxicon 2022, 210, 109–114. 10.1016/j.toxicon.2022.02.016. 35227741

[ref23] Brera C.; Debegnach F.; De Santis B.; Pannunzi E.; Berdini C.; Prantera E.; Gregori E.; Miraglia M. Simultaneous Determination of Aflatoxins and Ochratoxin A in Baby Foods and Paprika by HPLC with Fluorescence Detection: A Single-Laboratory Validation Study. Talanta 2011, 83, 1442–1446. 10.1016/j.talanta.2010.11.031. 21238734

[ref24] Ezekiel C. N.; Ortega-Beltran A.; Oyedeji E. O.; Atehnkeng J.; Kössler P.; Tairu F.; Hoeschle-Zeledon I.; Karlovsky P.; Cotty P. J.; Bandyopadhyay R. Aflatoxin in Chili Peppers in Nigeria: Extent of Contamination and Control Using Atoxigenic *Aspergillus flavus* Genotypes as Biocontrol Agents. Toxins 2019, 11, 429 10.3390/toxins11070429. PMC6669588 31336571

[ref25] RASSF. The Rapid Alert System for Food and Feed, European Commission. Eur. Comm. 2021. https://webgate.ec.europa.eu/rasff-window/screen/search.

[ref26] Perrone G.; Ferrara M.; Medina A.; Pascale M.; Magan N. Toxigenic Fungi and Mycotoxins in a Climate Change Scenario: Ecology, Genomics, Distribution, Prediction and Prevention of the Risk. Microorganisms 2020, 8, 1496 10.3390/microorganisms8101496. PMC7601308 33003323

[ref27] Huang Q.; Jiang K.; Tang Z.; Fan K.; Meng J.; Nie D.; Zhao Z.; Wu Y.; Han Z. Exposure Assessment of Multiple Mycotoxins and Cumulative Health Risk Assessment: A Biomonitoring-Based Study in the Yangtze River Delta, China. Toxins 2021, 13, 103 10.3390/toxins13020103. 33535530 PMC7912756

[ref28] EC. European Commission European Commission Regulation (EU) No 165/ 2010 of 26 February 2006 Amending Regulation (EC) No. 1881/2006 Setting Maximum Levels for Certain Contaminants in Foodstuffs as Regards Aflatoxins Off. J. Eur. Union L, 2010. https://eur-lex.europa.eu/LexUriServ/LexUriServ.do?uri=OJ:L:2010:050:0008:0012:EN:PDF.

[ref29] EC. European Commission Commission Regulation (EU) 2015/1137 of 13 July 2015 Amending Regulation (EC) No 1881-2006 as Regards the Maximum Level of Ochratoxin A in Capsicum spp. Spices. Off. J. Eur. Union L, 2015. https://eur-lex.europa.eu/legal-content/EN/TXT/PDF/?uri=CELEX:32015R1137&from=DE.

[ref30] Özbey F.; Kabak B. Natural Co-Occurrence of Aflatoxins and Ochratoxin A in Spices. Food Control 2012, 28, 354–361. 10.1016/j.foodcont.2012.05.039.

[ref31] Wan Ainiza W. M.; Jinap S.; Sanny M. Simultaneous Determination of Aflatoxins and Ochratoxin A in Single and Mixed Spices. Food Control 2015, 50, 913–918. 10.1016/j.foodcont.2014.10.051.

[ref32] Casquete R.; Rodríguez A.; Hernández A.; Martín A.; Bartolomé T.; Córdoba J. J.; Córdoba M. G. Occurrence of Toxigenic Fungi and Mycotoxins during Smoked Paprika Production. J. Food Prot. 2017, 80, 2068–2077. 10.4315/0362-028X.JFP-17-164. 29154716

[ref33] Hernández Hierro J. M.; Garcia-Villanova R. J.; Torrero P. R.; Fonseca I. M. T. Aflatoxins and Ochratoxin a in Red Paprika for Retail Sale in Spain: Occurrence and Evaluation of a Simultaneous Analytical Method. J. Agric. Food Chem. 2008, 56, 751–756. 10.1021/jf073002c. 18205310

[ref34] Set E.; Erkmen O. Occurrence of Aflatoxins in Ground Red Chili Pepper and Pistachio Nut. Int. J. Food Prop. 2014, 17, 2322–2331. 10.1080/10942912.2013.800985.

[ref35] Tosun A.; Ozden S. Ochratoxin A in Red Pepper Flakes Commercialised in Turkey. Food Addit. Contam. Part B Surveill. 2016, 9, 46–50. 10.1080/19393210.2015.1121929. 26600093

[ref36] EC. European Commission. European Commission Regulation (EC) No 401/2006 of 23 February 2006 Laying down the Methods of Sampling and Analysis for the Official Control of the Levels of Mycotoxins in Foodstuffs Off. J. Eur. Union 2006 L 70 12 34 https://eur-lex.europa.eu/legal-content/EN/TXT/PDF/?uri=CELEX:32006R0401&from=EN.

[ref37] Stroka J.; Anklam E.; Jörissen U.; Gilbert J. Immunoaffinity Column Cleanup with Liquid Chromatography Using Post-Column Broamination for Determination of Aflatoxins in Peanut Butter, Pistachio Paste, Fig Paste, and Paprika Powder: Collaborative Study. J. AOAC Int. 2000, 83, 320–340. 10.1093/jaoac/83.2.320. 10772170

[ref38] Trucksess M.; Weaver C.; Oles C.; D’Ovidio K.; Rader J. Determination of Aflatoxins and Ochratoxin A in Ginseng and Other Botanical Roots by Immunoaffinity Column Cleanup and Liquid Chromatography. J AOAC Int. 2006, 89, 624–630. 10.1093/jaoac/89.3.624. 16792061 PMC2586108

[ref39] Costa J.; Rodríguez R.; Garcia-Cela E.; Medina A.; Magan N.; Lima N.; Battilani P.; Santos C. Overview of Fungi and Mycotoxin Contamination in *Capsicum* Pepper and in Its Derivatives. Toxins 2019, 11, 27 10.3390/toxins11010027. PMC6356975 30626134

[ref40] Duman A. D. Storage of Red Chili Pepper under Hermetically Sealed or Vacuum Conditions for Preservation of Its Quality and Prevention of Mycotoxin Occurrence. J. Stored Prod. Res. 2010, 46, 155–160. 10.1016/j.jspr.2010.02.002.

[ref41] Atasoy A. F.; Hayoğlu İ.; Korkmaz A.; Kara E.; Yildirim A. Geleneksel Ev Isot Baharatının Aflatoksin Içeriğinin Belirlenmesi Üzerine Bir Araştırma. Harran Tarım ve Gıda Bilim. Derg. 2017, 21, 35–40. 10.29050/harranziraat.303132.

[ref42] Iqbal S. Z.; Mumtaz A.; Mahmood Z.; Waqas M.; Ghaffar A.; Ismail A.; Pervaiz W. Assessment of Aflatoxins and Ochratoxin a in Chili Sauce Samples and Estimation of Dietary Intake. Food Control 2021, 121, 107621 10.1016/j.foodcont.2020.107621.

[ref43] TFC. Regulation No. 2011/28157 the Maximum Allowed Level of Food Contaminants; Official Gazette of Publication: Ankara, Turkey, 2011, Vol. 28157. http://www.resmigazete.gov.tr.

[ref44] Ham H.; Kim S.; Kim M.-H.; Lee S.; Hong S. K.; Ryu J.; Lee T. Mycobiota of Ground Red Pepper and Their Aflatoxigenic Potential. J. Microbiol. 2016, 54, 832–837. 10.1007/s12275-016-6480-2. 27888464

[ref45] Set E.; Erkmen O. The Aflatoxin Contamination of Ground Red Pepper and Pistachio Nuts Sold in Turkey. Food Chem. Toxicol. 2010, 48, 2532–2537. 10.1016/j.fct.2010.06.027. 20600537

[ref46] Bircan C. The Determination of Aflatoxins in Spices by Immunoaffinity Column Extraction Using HPLC. Int. J. Food Sci. Technol. 2005, 40, 929–934. 10.1111/j.1365-2621.2005.01025.x.

[ref47] Jalili M.; Jinap S. Natural Occurrence of Aflatoxins and Ochratoxin A in Commercial Dried Chili. Food Control 2012, 24, 160–164. 10.1016/j.foodcont.2011.09.020.

[ref48] Marín S.; Colom C.; Sanchis V.; Ramos A. J. Modelling of Growth of Aflatoxigenic *A. flavus* Isolates from Red Chilli Powder as a Function of Water Availability. Int. J. Food Microbiol. 2009, 128, 491–496. 10.1016/j.ijfoodmicro.2008.10.020. 19046614

[ref49] Erdogan A. The Aflatoxin Contamination of Some Pepper Types Sold in Turkey. Chemosphere 2004, 56, 321–325. 10.1016/j.chemosphere.2004.02.020. 15183993

[ref50] Cho S. H.; Lee C. H.; Jang M. R.; Son Y. W.; Lee S. M.; Choi I. S.; Kim S. H.; Kim D. B. Aflatoxins Contamination in Spices and Processed Spice Products Commercialized in Korea. Food Chem. 2008, 107, 1283–1288. 10.1016/j.foodchem.2007.08.049.

[ref51] Huff W. E.; Kubena L. F.; Harvey R. B.; Doerr J. A. Mycotoxin Interactions in Poultry and Swine. J. Anim. Sci. 1988, 66, 2351–2355. 10.2527/jas1988.6692351x. 3170377

[ref52] Smith M.-C. C.; Madec S. S. S.; Coton E.; Hymery N. Natural Co-Occurrence of Mycotoxins in Foods and Feeds and Their *in Vitro* Combined Toxicological Effects. Toxins 2016, 8, 94 10.3390/toxins8040094. 27023609 PMC4848621

[ref53] Sedmíková M.; Reisnerová H.; Dufková Z.; Bárta I.; Jílek F. Potential Hazard of Simultaneous Occurrence of Aflatoxin B_1_ and Ochratoxin A. Vet. Med. 2001, 46, 169–171. 10.17221/7876-VETMED.

[ref54] EFSA. European Food Safety Authority. Risk Assessment of Aflatoxins in Food 2020, 18 (3), 6040 https://efsa.onlinelibrary.wiley.com/doi/full/10.2903/j.efsa.2020.6040. 10.2903/j.efsa.2020.6040 PMC7447885 32874256

[ref55] JECFA. The Joint FAO/WHO Expert Committee on Food Additives. Evaluation of Certain Food Additives and Contaminants: Sixty-Eighth Report of the Joint FAO/WHO Expert Committee on Food Additive. WHO Technical Report Series No 947; WHO: Geneva, Switzerland, 2007pp 169-180.

[ref56] EFSA. Opinion of the Scientific Panel on Contaminants in the Food Chain on a Request from the Commission Related to Ochratoxin A in Food EFSA J 2006 365 1 56 https://www.efsa.europa.eu/en/efsajournal/pub/365.

[ref57] Yogendrarajah P.; Jacxsens L.; Lachat C.; Walpita C. N.; Kolsteren P.; De Saeger S.; De Meulenaer B. Public Health Risk Associated with the Co-Occurrence of Mycotoxins in Spices Consumed in Sri Lanka. Food Chem. Toxicol. 2014, 74, 240–248. 10.1016/j.fct.2014.10.007. 25455891

[ref58] Sakin F.; Tekeli İO.; Yipel M.; Kürekci C. Occurrence and Health Risk Assessment of Aflatoxins and Ochratoxin a in *Sürk*, a Turkish Dairy Food, as Studied by HPLC. Food Control 2018, 90, 317–323. 10.1016/j.foodcont.2018.03.012.

[ref59] Kilic S.; Cam I. B.; Tongur T.; Kilic M. Health Risk Assessment of Exposure to Heavy Metals and Aflatoxins via Dietary Intake of Dried Red Pepper from Marketplaces in Antalya, Southern Turkey. J. Food Sci. 2018, 83, 2675–2681. 10.1111/1750-3841.14322. 30178501

[ref60] Kulahi A.; Kabak B. A Preliminary Assessment of Dietary Exposure of Ochratoxin A in Central Anatolia Region, Turkey. Mycotoxin Res. 2020, 36, 327–337. 10.1007/s12550-020-00397-6. 32621108

[ref61] Oktay Basegmez H. I. Dietary Exposure Assessment of Aflatoxin from Dried Figs in Turkey. Hittite J. Sci. Eng. 2019, 6, 173–177. 10.17350/hjse19030000144.

[ref62] Heshmati A.; Zohrevand T.; Khaneghah A. M.; Mozaffari Nejad A. S.; Sant’Ana A. S. Co-Occurrence of Aflatoxins and Ochratoxin A in Dried Fruits in Iran: Dietary Exposure Risk Assessment. Food Chem. Toxicol. 2017, 106, 202–208. 10.1016/j.fct.2017.05.046. 28552785

[ref63] Huong B. T. M.; Tuyen L. D.; Tuan D. H.; Brimer L.; Dalsgaard A. Dietary Exposure to Aflatoxin B_1_, Ochratoxin A and Fuminisins of Adults in Lao Cai Province, Viet Nam: A Total Dietary Study Approach. Food Chem. Toxicol. 2016, 98, 127–133. 10.1016/j.fct.2016.10.012. 27746326

[ref64] Kabak B. Aflatoxins in Hazelnuts and Dried Figs: Occurrence and Exposure Assessment. Food Chem. 2016, 211, 8–16. 10.1016/j.foodchem.2016.04.141. 27283601

[ref65] Bol E. K.; Araujo L.; Veras F. F.; Welke J. E. Estimated Exposure to Zearalenone, Ochratoxin A and Aflatoxin B_1_ through the Consume of Bakery Products and Pasta Considering Effects of Food Processing. Food Chem. Toxicol. 2016, 89, 85–91. 10.1016/j.fct.2016.01.013. 26807886

[ref66] Ojuri O. T.; Ezekiel C. N.; Eskola M. K.; Šarkanj B.; Babalola A. D.; Sulyok M.; Hajšlová J.; Elliott C. T.; Krska R. Mycotoxin Co-Exposures in Infants and Young Children Consuming Household- and Industrially-Processed Complementary Foods in Nigeria and Risk Management Advice. Food Control 2019, 98, 312–322. 10.1016/j.foodcont.2018.11.049.

[ref67] Trucksess M. W.; Weaver C. M.; Oles C. J.; Fry F. S.; Noonan G. O.; Betz J. M.; Rader J. I. Determination of Aflatoxins B_1_, B_2_, G_1_, and G_2_ and Ochratoxin A in Ginseng and Ginger by Multitoxin Immunoaffinity Column Cleanup and Liquid Chromatographic Quantitation: Collaborative Study. J. AOAC Int. 2008, 91, 511–523. 10.1093/jaoac/91.3.511. 18567295 PMC2586123

[ref68] Eurachem. The Fitness for the Purpose of Analytical Methods. A Laboratory Guide to Method Validation and Related Topics. Middlesex, TW110LY, United Kingdom: Eurachem Working Group, 2014. https://www.eurachem.org/images/stories/Guides/pdf/MV_guide_2nd_ed_EN.pdf.

[ref69] EFSA. European Food Safety Authority. Opinion of the Scientific Committee on a Request from EFSA Related to a Harmonized Approach for Risk Assessment of Substances Which Are Both Genotoxic and Carcinogenic EFSA J 2005 282 1 31 https://www.efsa.europa.eu/en/efsajournal/pub/282.

[ref70] Scientific Report of EFSA: Management of Left-Censored Data in Dietary Exposure Assessment of Chemical Substances. EFSA J. 2010, 8, 1–96. 10.2903/j.efsa.2010.1557.

[ref71] WHO. World Health Organization. Prevention of Mother-to-Child Transmission of Hepatitis B Virus: Guidelines on Antiviral Prophylaxis in Pregnancy: Web Annex A: Systematic Review of the Efficacy and Safety of Antiviral Therapy during Pregnancy. 2020. https://apps.who.int/iris/bitstream/handle/10665/333391/9789240002708-eng.pdf. 32833415

[ref72] Mehmet D.; Meliksah E.; Serif Y.; Gunay S.; Tuncer Ö.; Zeynep S. Prevalence of Hepatitis B Infection in the Southeastern Region of Turkey: Comparison of Risk Factors for HBV Infection in Rural and Urban Areas. Jpn. J. Infect. Dis. 2005, 58, 15–19. 15728984

[ref73] JECFA. The Joint FAO/WHO Expert Committee on Food Additives. Eighty-Third Report of the Joint FAO/WHO Expert Committee on Food Additives. Evaluation of Certain Contaminants in Food,. WHO Technical Report Series, 2017, pp 1-166.

[ref74] SCF. The European Union Scientific Committee for Food. European Commission DG XXIV Unit B3, Thirty-Fifth Report. Opinion on Aflatoxins B1, B2, G1, G2, M1, and Patulin, 1994.

[ref75] JECFA. The Joint FAO/WHO Expert Committee on Food Additives. Evaluation of Certain Food Additives and Contaminants: Fifty-Fifth Report of the Joint FAO/WHO Expert Committee on Food Additives, WHO Technical Report Series No 901, WHO: Geneva, Switzerland; 2001.

[ref76] Kuiper-Goodman T. Food Safety: Mycotoxins and Phycotoxins in Perspective. In Mycotoxins and Phycotoxins: Developments in Chemistry, Toxicology and Food Safety.Alakens, Fort Collins; Miraglia M.; van Egmond H. P.; Brera C.; Gilbert J., Eds.; 1998; pp 25–48.

