# Supplementary material for: Simultaneous Detection of Ochratoxin A and Aflatoxins in Industrial and Traditional Red and isot Pepper Flakes along with Dietary Exposure Risk Assessment
Source: ACS Omega. 2022 Aug 26;7(36):31756–66. doi: 10.1021/acsomega.2c02236 (PMC9476180; doi:10.1021/acsomega.2c02236)
Supplement: Supplementary file 1 — ao2c02236_si_001.pdf [file ao2c02236_si_001.pdf]

Supporting Information

Simultaneous detection of ochratoxin A and aflatoxins in industrial and traditional red and *isot* pepper flakes along with dietary exposure risk assessment

Sebahat Oztekin[1,2], and Funda Karbancioglu-Guler[1*]

[1]Department of Food Engineering, Faculty of Chemical and Metallurgical Engineering, Istanbul Technical University, Maslak 34469, Istanbul, Turkey.

[2]Department of Food Engineering, Faculty of Engineering, Bayburt University, Bayburt 69000, Turkey.

Corresponding author

Funda Karbancioglu-Guler, karbanci@itu.edu.tr

Department of Food Engineering, Faculty of Chemical and Metallurgical Engineering, Istanbul Technical University, Maslak 34469, Istanbul, Turkey.

a

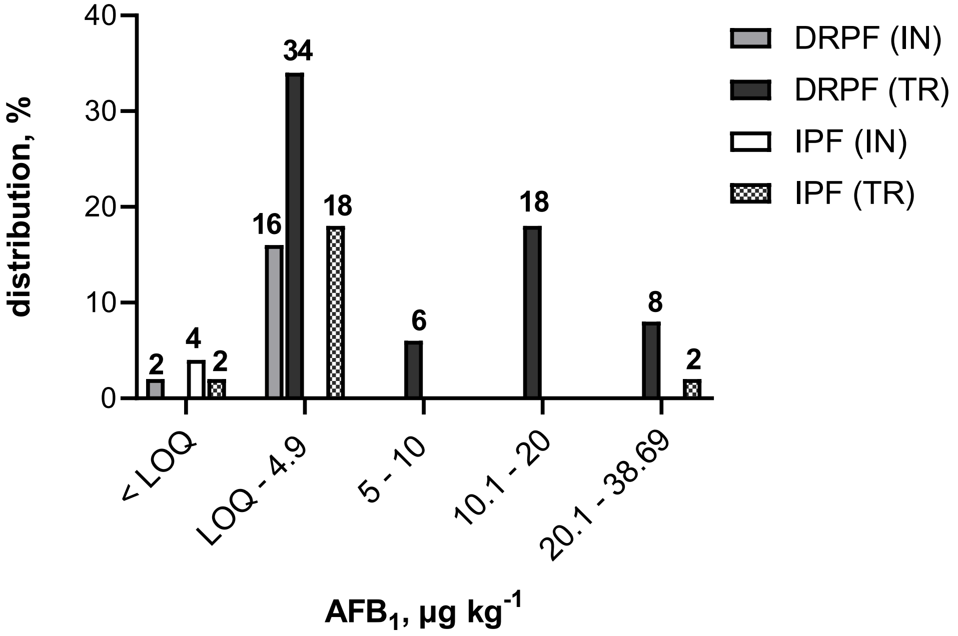

DRPF (IN)
DRPF (TR)
IPF (IN)
IPF (TR)
distribution, %
40
30
20
10
0
2
4
2
16
34
18
6
18
8
2
< LOQ
LOQ - 4.9
5 - 10
10.1 - 20
20.1 - 38.69
AFB1, µg kg-1

b

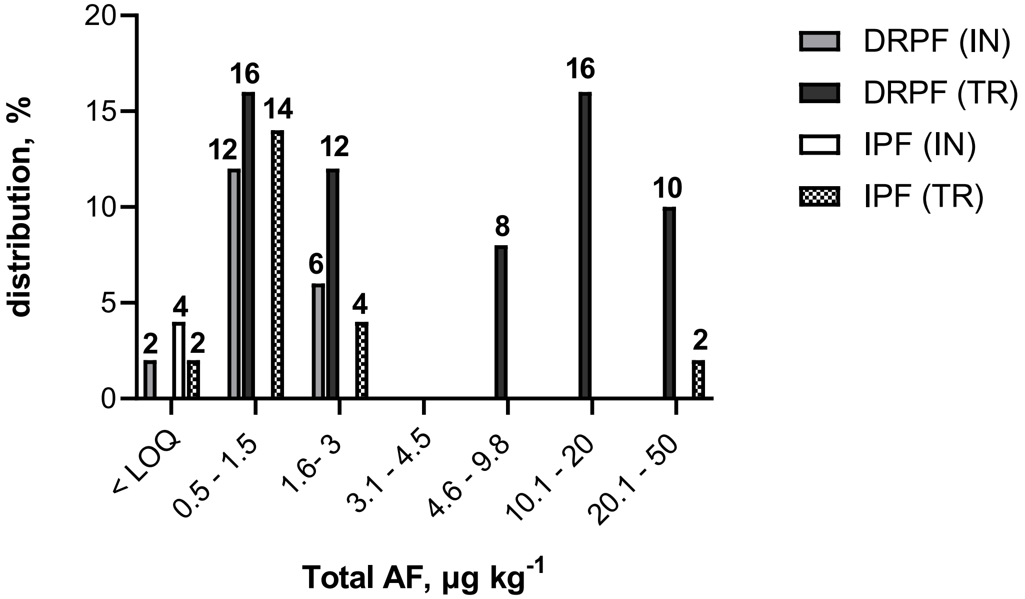

DRPF (IN)
DRPF (TR)
IPF (IN)
IPF (TR)
distribution, %
20
15
10
5
0
2
4
2
12
16
14
6
12
4
8
16
10
2
< LOQ
0.5 - 1.5
1.6- 3
3.1 - 4.5
4.6 - 9.8
10.1 - 20
20.1 - 50
Total AF, µg kg-1

c

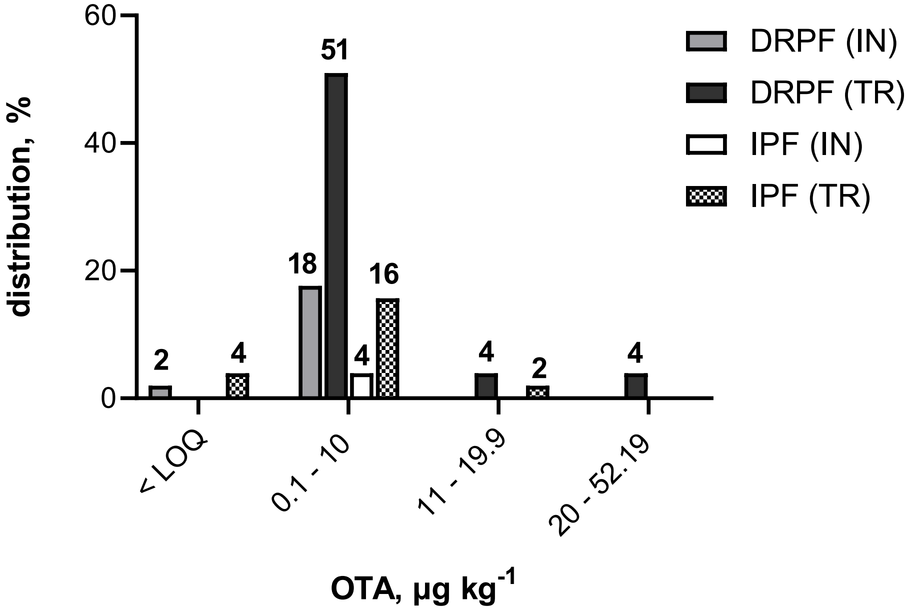

Figure. S1. AFB_1 (A), Total AF (B), and OTA (C) contamination ranges in red pepper samples

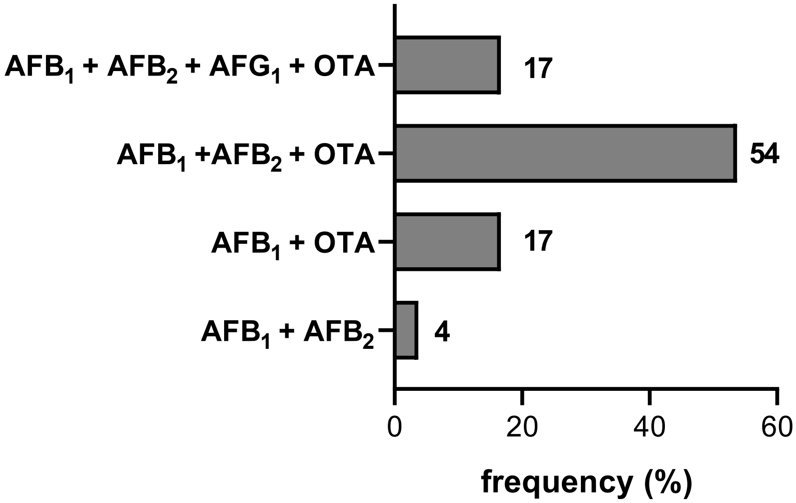

Figure. S2. Co-occurrence of AFs and OTA in samples
